# Evaluation of the cultural, health-related, and structural barriers to blood donation among women in Saudi Arabia: a cross-sectional study

**DOI:** 10.3389/fpubh.2026.1806754

**Published:** 2026-04-15

**Authors:** Abdulrahman Alshalani, Turky AlSulaiman, Judy Alharisy, Yazan Alqahtani, Abdulmajeed Altihani, Haifa AlNafea

**Affiliations:** Department of Clinical Laboratory Sciences, College of Applied Medical Sciences, King Saud University, Riyadh, Saudi Arabia

**Keywords:** blood donation, cultural and health-related barriers, donation attitudes and beliefs, female blood donors, women's health

## Abstract

**Background:**

Blood transfusion is a critical component of healthcare systems worldwide, yet maintaining adequate blood supplies remains challenging, particularly when voluntary donation rates are low. In Saudi Arabia, women are markedly underrepresented among blood donors despite national efforts to promote donation. Understanding the cultural, social, and physiological factors influencing women's participation is essential for developing effective, gender-sensitive strategies.

**Methods:**

A cross-sectional, online survey was conducted among Saudi women aged 18–65 years between April and September 2025. The questionnaire assessed sociodemographic characteristics, knowledge, attitudes, health-related concerns, and accessibility related to blood donation. Descriptive statistics and nonparametric tests were used to analyze data from 503 respondents.

**Results:**

Although more than 85% of participants considered blood donation a social responsibility, only 15.1% reported a history of donation, indicating a clear attitude–behavior gap. Health-related uncertainty was prominent, with 47.7% avoiding donation due to uncertainty about eligibility and 24.1% reporting previous deferral for health reasons. Cultural misconceptions, particularly regarding menstruation and female health, were common. Structural barriers were also evident; nearly half cited lack of time, while willingness to donate increased substantially if mobile donation units were available (74.2%).

**Conclusion:**

Saudi women's low participation in blood donation is driven by the interaction of health-related uncertainty, cultural misconceptions, and limited accessibility rather than lack of altruistic motivation. Addressing these barriers through culturally sensitive education, clear communication about eligibility, and improved access—especially via mobile and community-based donation services—may help convert willingness into sustained donor participation and strengthen the national blood supply.

## 1 Introduction

Blood transfusion is a cornerstone of modern healthcare, supporting a wide range of clinical services including major surgery, trauma management, oncology, obstetric care, and the treatment of chronic diseases (1, 2). The sustainability of transfusion services relies almost entirely on voluntary blood donation, making donor recruitment and retention critical public health priorities. Despite global efforts to strengthen blood donation systems, many countries continue to experience supply shortages that fail to meet increasing clinical demand (3). In Saudi Arabia, maintaining an adequate blood supply remains a persistent challenge. Although substantial investments have been made to improve blood bank infrastructure and promote donation through national awareness campaigns, demand continues to outpace supply. In 2020 alone, more than 300,000 voluntary blood donations were reported nationwide; however, rapid population growth and expansion of healthcare services have intensified pressure on blood reserves (4, 5). Addressing this gap requires not only increasing overall donation rates but also engaging underrepresented segments of the population, particularly women, whose contribution to blood donation remains disproportionately low.

Globally, women constitute a substantial proportion of blood donors, and several countries have achieved near parity between male and female participation (3). In contrast, Saudi Arabia is among a small group of countries in which women contribute less than 10% of total blood donations (3, 6). This disparity is particularly striking given that women represent approximately half of the Saudi population (7). National data indicate that women account for as little as 2%−3% of registered blood donors in governmental centers, while hospital-based studies consistently report female participation rates below 7% (4, 8). These figures diverge sharply from international trends and suggest that female underrepresentation in Saudi Arabia cannot be explained by demographic distribution alone.

Previous studies conducted within the Kingdom have attempted to explore the factors underlying this gender gap and have identified a complex interplay of physiological, perceptual, cultural, and structural barriers. Physiologically, low hemoglobin levels and iron deficiency are the most common causes of donor deferral among women, with female donors reported to be several times more likely than males to be deferred for this reason (6, 9). Structurally, limited access to donation sites, scarcity of mobile blood drives, time constraints, and competing family or work responsibilities have been repeatedly cited as deterrents, particularly among students and working women (10). Perceptually, misconceptions surrounding menstruation, pregnancy, lactation, anemia, and post-donation weakness persist, leading many women to self-exclude from donation or to interpret temporary deferrals as permanent ineligibility (11–13).

Social and cultural factors further compound these barriers. Blood donation campaigns are often conducted in environments perceived as male-dominated or insufficiently accommodating to women, which may discourage participation (14, 15). Although restrictions on women's mobility have eased substantially in recent years, female donation rates have not increased proportionally, suggesting that reduced mobility alone does not fully explain low participation. Instead, persistent misperceptions regarding health eligibility, coupled with limited accessibility and high deferral rates, appear to play a central role. Importantly, much of the existing evidence is derived from single-center or university-based studies with limited sample sizes, restricting the generalizability of findings and the development of evidence-based, gender-sensitive interventions (12).

Despite growing interest in blood donation practices in Saudi Arabia, comprehensive population-based studies examining the combined cultural, social, and physiological determinants of women's participation remain scarce. As a result, the mechanisms driving female underrepresentation are not fully understood, and policy and intervention efforts lack robust empirical guidance. Therefore, the present cross-sectional study aimed to identify and evaluate the cultural, social, and physiological barriers to blood donation among Saudi women aged 18–65 years. By examining knowledge, attitudes, health-related concerns, and accessibility factors, this study seeks to clarify the determinants of low female participation and to inform targeted strategies that can translate women's willingness to donate into sustained engagement with the national blood donation system.

## 2 Methods

### 2.1 Study design and participants

This cross-sectional, survey-based study was conducted among Saudi females aged 18–65 years between April and September 2025. Data were collected using an anonymous online questionnaire (Microsoft Forms) distributed via social media platforms (WhatsApp). Participants accessed the survey directly through the shared link, provided electronic informed consent, and completed the questionnaire voluntarily. Eligible participants included Saudi females within the target age range who consented and completed the questionnaire. Females outside the specified age range and those reporting permanent medical conditions that contraindicate blood donation were excluded. Based on a 95% confidence level, 5% margin of error, and an assumed population proportion of 50%, the minimum required sample size was 384 participants. To enhance statistical power and enable subgroup analyses, a larger sample was targeted. Of the 736 responses received, 233 were excluded due to age ineligibility, reported chronic health conditions, or incomplete responses, resulting in a final analytic sample of 503 participants. The study protocol was reviewed and approved by the Institutional Review Board of King Saud University (Approval No. KSU-HE-25-551, April 2025).

### 2.2 Questionnaire

Data were collected using a structured questionnaire adapted from previously validated instruments (16, 17). The questionnaire comprised 45 items organized into five sections: eligibility and sociodemographic characteristics (nine items), which captured information on age, marital status, number of children, region of residence, educational level, occupation, and household income; knowledge of blood donation (11 items), which assessed participants' understanding of donation eligibility, procedures, and health-related issues, with correct responses scored as 1 and incorrect responses as 0, yielding a total knowledge score ranging from 0 to 11; attitudes and beliefs (11 items), which evaluated perceptions, fears, social influences, and motivational beliefs using a five-point Likert scale ranging from strongly agree to strongly disagree; health-related concerns (eight items), which explored perceived health barriers, prior deferrals, and concerns related to donation safety and eligibility; and accessibility and willingness to donate (seven items), which assessed logistical barriers, perceived accessibility of donation centers, and willingness to donate under improved conditions. The full questionnaire is provided in the Supplementary material.

### 2.3 Data management and statistical analysis

Data were exported from Microsoft Forms into Microsoft Excel and analyzed using IBM SPSS Statistics version 25 (IBM Corp., Armonk, NY, USA). Graphical representations were generated using GraphPad Prism version 9.4.1 (GraphPad Software, San Diego, CA, USA). Categorical variables were summarized as frequencies and percentages, while continuous variables were described using medians and interquartile ranges (IQR), as appropriate. Associations between categorical variables, including blood donation history and sociodemographic characteristics, were examined using Pearson's chi-squared test. Comparisons of knowledge scores between two independent groups were conducted using the Mann–Whitney *U*-test, while comparisons across more than two groups were performed using the Kruskal–Wallis test, followed by Bonferroni-adjusted *post hoc* analyses where applicable. A *p*-value < 0.05 was considered statistically significant for all analyses.

## 3 Results

### 3.1 Participant characteristics

A total of 503 women were included in the analysis, with no missing data across the studied variables. The sociodemographic characteristics of the study population are summarized in Table 1. Briefly, the largest proportion of participants were aged 18–25 years (34.8%), followed by those aged 36–45 years (25.8%). Slightly more than half of the participants were single (51.3%), and 52.3% reported having no children. Most participants resided in the Central region (75.1%) and held a bachelor's degree (65.0%). Regarding employment status, (41.2%) were employed and (32.4%) were students. Monthly income was most commonly in the range of 10,000–20,000 SAR (34.0%).

**Table 1:** Sociodemographic characteristics of the study participants (*N* = 503).

Variable (*N* = 503)	*N* (%)
Age	
18–25	175 (34.8)
26–35	78 (15.5)
36–45	130 (25.8)
46–55	71 (14.1)
More than 55	49 (9.7)
Marital status	
Single	258 (51.3)
Married	245 (48.7)
Number of children	
0	263 (52.3)
1–2	62 (12.3)
3–4	100 (19.9)
More than 4	78 (15.5)
Region of residence	
Central	378 (75.1)
Western	72 (14.3)
Eastern	20 (4.0)
Northern	9 (1.8)
Southern	24 (4.8)
Educational level	
Uneducated	2 (0.4)
High school	105 (20.9)
Bachelor's degree	327 (65.0)
Postgraduate degree	69 (13.7)
Occupation	
Student	163 (32.4)
Employed	207 (41.2)
Self–employed	8 (1.6)
Unemployed	125 (24.9)
Household income	
Less than 5,000 SAR	55 (10.9)
5,000–10,000 SAR	141 (28.0)
10,000–20,000 SAR	171 (34.0)
More than 20,000 SAR	136 (27.0)

SAR, Saudi Riyal.

### 3.2 Donation history and sociodemographic characteristics

Overall, 76 of 503 women (15.1%) reported a history of blood donation. The association between donation history and sociodemographic characteristics is presented in Table 2. A significant association was observed between age group and donation history (χ^2^ = 14.51, p = 0.014). The highest proportion of donors was among women aged 36–45 years (30/76, 39.5%), followed by those aged 18–25 years (15/76, 19.7%). Women aged >55 years accounted for 9.2% (7/76) of donors. Furthermore, educational level was significantly associated with donation history (χ^2^ = 14.51, *p* = 0.002). Most donors held at least a bachelor's degree (50/76, 65.8%), while 25.0% (19/76) had a postgraduate degree. Donors with only high-school education represented 9.2% (7/76). A strong association was also found between occupation and donation history (χ^2^ = 23.78, *p* < 0.001). Employed women constituted the largest proportion of donors (48/76, 63.2%), followed by students (12/76, 15.8%) and unemployed participants (14/76, 18.4%). No statistically significant association was found between donation history and marital status, number of children, region, or household income.

**Table 2:** Association between sociodemographic characteristics and blood donation history among females.

Variable	History of blood donation		*p–value*
	Yes *N* = 76 (15.1%)	No *N* = 427 (84.9%)	
Age			0.014^*^
18–25	15 (3.0)	160 (31.8)	
26–35	12 (2.4)	66 (13.1)	
36–45	30 (6.0)	100 (19.9)	
46–55	12 (2.4)	59 (11.7)	
More than 55	7 (1.4)	42 (8.3)	
Marital status			0.321
Single	35 (7.0)	223 (44.3)	
Married	41 (8.2)	204 (40.6)	
Number of children			0.088
0	35 (7.0)	228 (45.3)	
1–2	16 (3.2)	46 (9.1)	
3–4	13 (2.6)	87 (17.3)	
More than 4	12 (2.4)	66 (13.1)	
Region of residence			0.055
Central	66 (13.1)	312 (62.0)	
Western	6 (1.2)	66 (13.1)	
Eastern	2 (0.4)	18 (3.6)	
Northern	2 (0.4)	7 (1.4)	
Southern	0 (0)	24 (4.8)	
Educational level			0.002^**^
Uneducated	0 (0)	2 (0.4)	
High school	7 (1.4)	98 (19.5)	
Bachelor's degree	50 (9.9)	277 (55.1)	
Postgraduate degree	19 (3.8)	50 (9.9)	
Occupation			< 0.001^***^
Student	12 (2.4)	151 (30.0)	
Employed	48 (9.5)	159 (31.6)	
Self–employed	2 (0.4)	6 (1.2)	
Unemployed	14 (2.8)	111 (22.1)	
Household income			0.467
Less than 5,000 SAR	5 (1.0)	50 (9.9)	
5,000–10,000 SAR	23 (4.6)	118 (23.5)	
10,000–20,000 SAR	24 (4.8)	147 (29.2)	
More than 20,000 SAR	24 (4.8)	112 (22.3)	

SAR, Saudi Riyal.

Associations were assessed using Pearson's chi-squared test.

^*^p < 0.05; ^**^p < 0.01; ^***^p < 0.001.

### 3.3 Knowledge score according to sociodemographic characteristics

**Figure 1:**
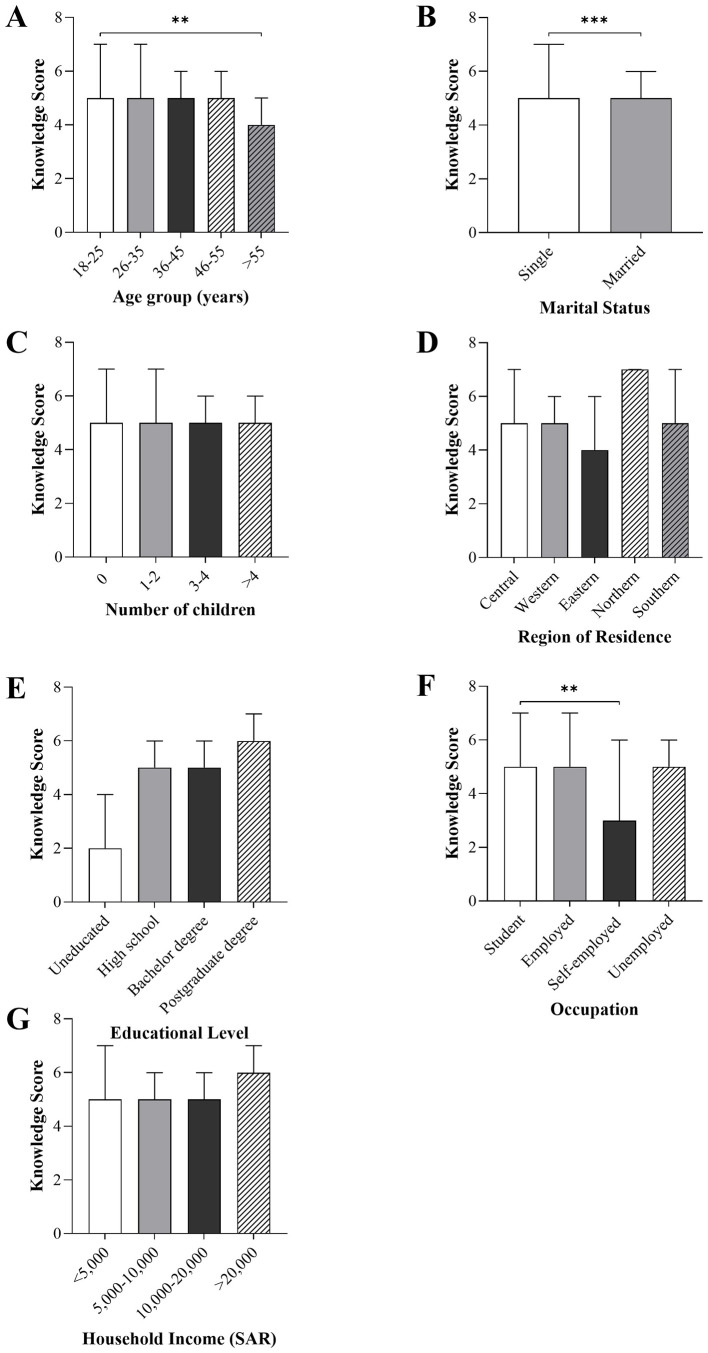
Differences in blood donation knowledge scores according to sociodemographic characteristics. knowledge scores were compared according to **(A)** age group, **(B)** marital status, **(C)** number of children, **(D)** region of residence, **(E)** educational level, **(F)** occupation, and **(G)** household income. Bars represent the median with upper quartile (Q3). Group comparisons were performed using the Kruskal–Wallis test for variables with more than two categories **(A, C–G)** and the Mann–Whitney *U*-test for two-group comparisons **(B)**. **p < 0.01; ***p < 0.001.

Knowledge scores differed significantly across several sociodemographic characteristics, as illustrated in Figure 1. A significant variation was observed across age groups (*p* < 0.001), with participants aged >55 years having the lower mean knowledge score compared to those aged 18–25 years. Married women demonstrated higher knowledge scores than single women (*p* < 0.001). Knowledge scores also differed significantly by occupation (*p* < 0.001), with self-employed participants showing lower scores compared with students. No significant differences in knowledge scores were found according to the number of children, region of residence, educational level, or household income.

### 3.4 Attitudes and beliefs of females toward blood donation

Table 3 summarizes females' attitudes and beliefs toward blood donation. The majority of participants did not perceive fear-related factors as major barriers, with (59.2%) disagreeing that fear of needles influences their decision and (66.8%) disagreeing that fear of seeing blood is discouraging. Similarly, (63.2%) disagreed that fear of weakness or illness prevents them from donating, while (24.1%) reported such concerns. Time-related constraints were more prominent, with (46.3%) identifying lack of time as a barrier to donation. Knowledge-related factors were also evident, as (38.8%) agreed that they had never donated because they lacked sufficient information, whereas (53.9%) reported having no specific reason for not donating. Regarding physiological beliefs, (46.5%) agreed that females should not donate blood during menstruation and (34.8%) expressed neutral views, while only (20.1%) believed that females of childbearing age should avoid blood donation and (52.1%) disagreed with this statement. Social influence appeared limited, with only (16.1%) reporting that family or friends affect their decision, compared with (70.8%) who disagreed. Notably, positive attitudes toward donation were highly prevalent, as (86.3%) of participants agreed that blood donation is a social responsibility and (85.6%) indicated that they are likely to encourage others to donate blood.

**Table 3:** Attitudes and beliefs toward blood donation among females.

Attitudes and beliefs	Strongly agree	Agree	Neutral	Disagree	Strongly disagree
Fear of needles prevents me from donating blood	50 (9.9)	70 (13.9)	85 (16.9)	136 (27.0)	162 (32.2)
Fear of seeing blood discourages me from donating	35 (7.0)	58 (11.5)	74 (14.7)	163 (32.4)	173 (34.4)
I avoid donating blood because I'm afraid it will make me weak or sick	29 (5.8)	92 (18.3)	64 (12.7)	151 (30.0)	167 (33.2)
Lack of time is the main reason I do not donate blood	76 (15.1)	157 (31.2)	87 (17.3)	112 (22.3)	71 (14.1)
I have never donated blood because I don't know enough about it	73 (14.5)	122 (24.3)	85 (16.9)	115 (22.9)	108 (21.5)
There is no specific reason why I haven't donated blood	97 (19.3)	174 (34.6)	84 (16.7)	81 (16.1)	67 (13.3)
Females should not donate blood during menstruation	88 (17.5)	146 (29.0)	175 (34.8)	61 (12.1)	33 (6.6)
Females of childbearing age should avoid donating blood	36 (7.2)	65 (12.9)	140 (27.8)	153 (30.4)	109 (21.7)
My family or friends influence my decision whether to donate blood	20 (4.0)	61 (12.1)	66 (13.1)	174 (34.6)	182 (36.2)
Blood donation is a social responsibility	235 (46.7)	199 (39.6)	44 (8.7)	16 (3.2)	9 (1.8)
I am likely to encourage others to donate blood	221 (43.9)	210 (41.7)	59 (11.7)	8 (1.6)	5 (1.0)

### 3.5 Health concerns of females toward blood donation

Table 4 summarizes females' health-related concerns regarding blood donation. Nearly one-quarter of participants (24.1%) reported that they had previously been informed that they were ineligible to donate due to health reasons, while (19.1%) indicated having a current health condition that makes them hesitant to donate. Concerns regarding donation-related side effects were generally low, with (35.4%) reporting no concern and (31.6%) reporting only slight concern. However, a substantial proportion of participants (40.3%) expressed concern that blood donation might worsen existing health conditions. Uncertainty regarding health eligibility was common, as (47.7%) agreed that they avoid donating because they are unsure whether they meet health requirements. Additionally, (35.6%) agreed with the perception that most females are not healthy enough to donate blood regularly. Despite these concerns, willingness to donate improved markedly with better accessibility, as (74.2%) reported being more willing to donate if a mobile donation unit were available (Supplementary Table 1).

**Table 4:** Health-related concerns toward blood donation among females.

Health concerns	*N* (%)
Have you ever been told that you were not eligible to donat	
blood due to health reasons?	
Yes	121 (24.1)
No	382 (75.9)
Do you currently have any health condition that makes you	
hesitant to donate blood?	
Yes	96 (19.1)
No	407 (80.9)
How concerned are you about experiencing side effects	
(e.g., dizziness, fatigue) after donating blood?	
Not at all concerned	178 (35.4)
Slightly concerned	159 (31.6)
Moderately concerned	98 (19.5)
Very concerned	41 (8.2)
Extremely concerned	27 (5.4)
I am worried that donating blood might worsen existing	
health conditions (such as anemia or low blood pressure).	
Strongly agree	50 (12.5)
Agree	140 (27.8)
Neutral	103 (20.5)
Disagree	106 (21.1)
Strongly disagree	104 (20.7)
I avoid donating blood because I'm not sure if my health	
status allows it.	
Strongly agree	63 (12.5)
Agree	177 (35.2)
Neutral	80 (15.9)
Disagree	95 (18.9)
Strongly disagree	88 (17.5)
I believe that most females are not healthy enough to donate	
blood regularly.	
Strongly agree	40 (8.0)
Agree	139 (27.6)
Neutral	107 (21.3)
Disagree	137 (27.2)
Strongly disagree	80 (15.9)

## 4 Discussion

This study examined cultural, social, and physiological factors influencing blood donation among Saudi women and provides important insight into the persistent underrepresentation of females in the national donor pool. Consistent with previous Saudi and regional studies, the findings demonstrate that low donation rates among women are not driven by negative attitudes or lack of altruism, but rather by health-related uncertainty, cultural misconceptions, and structural barriers that hinder the translation of willingness into actual donation behavior (10–12).

A central finding of this study is the pronounced discrepancy between attitudes and behavior. Although more than (85%) of participants considered blood donation a social responsibility, only (15.1%) had ever donated. This attitude–behavior gap has been repeatedly reported in Saudi Arabia, where favorable perceptions toward blood donation coexist with low participation rates, particularly among women (4, 10, 13). Similar patterns have also been observed internationally, including in Iran and Nigeria, suggesting that positive attitudes alone rarely translate into donation without enabling structural conditions (16, 17). The current findings further support behavioral models such as the Theory of Planned Behavior, which emphasize that intention must be accompanied by perceived control and opportunity for behavior to occur (18). The fact that more than half of non-donors reported no specific reason for not donating reinforces earlier observations that passive barriers, such as inconvenience or uncertainty, may be more influential than explicit refusal (11).

Sociodemographic differences in donation behavior observed in this study are also consistent with earlier research. Women aged 36–45 years, those with higher educational attainment, and employed participants were more likely to have donated blood, aligning with national data showing higher donation rates among older and economically active individuals (4, 8). In contrast, younger women demonstrated higher knowledge scores but lower donation rates, a finding previously reported among Saudi university students (10, 13). This knowledge–behavior mismatch suggests that awareness alone does not overcome logistical and psychosocial barriers, particularly among younger women who may perceive donation as less urgent or less compatible with their daily routines.

Health-related concerns emerged as a major barrier to donation and closely mirror findings from prior Saudi studies. Nearly one-quarter of participants reported previous deferral due to health reasons, and almost half avoided donation due to uncertainty about eligibility. Similar levels of health-related hesitation have been reported in Riyadh, Jeddah, and Najran, where fear of anemia, dizziness, or worsening health conditions were common deterrents among women (11, 12). Importantly, concerns about acute post-donation side effects were relatively low in this study, suggesting that perceived long-term health impact and eligibility uncertainty—rather than fear of the procedure itself—are more influential barriers. This aligns with evidence showing that hemoglobin-related deferral, the most frequent cause of female donor rejection, can discourage future donation attempts and promote self-deferral even when eligibility is restored (6, 8, 9).

Cultural misconceptions surrounding menstruation and female physiology were particularly prominent. Nearly half of participants believed that women should not donate blood during menstruation, a finding consistent with earlier studies among Saudi female students and community samples (13, 15). These beliefs reflect persistent associations between menstruation, blood loss, and physical weakness, which may be reinforced by high rates of hemoglobin-based deferral among women. Similar misconceptions have been reported in Middle Eastern and Asian contexts, indicating that these beliefs are culturally entrenched rather than medically grounded (16). Without targeted clarification, such misconceptions may normalize self-exclusion and reduce repeat donation among otherwise eligible women.

Structural and accessibility barriers identified in this study strongly align with existing literature. Almost half of participants cited lack of time as a barrier, and only half knew the location of the nearest donation center. These findings are consistent with Saudi studies showing that limited access to donation sites, inflexible schedules, and scarcity of mobile blood drives disproportionately affect women (10, 12, 15). Notably, willingness to donate increased markedly when accessibility was hypothetically improved, with more than (70%) indicating readiness to donate if mobile units were available. Similar improvements in willingness following increased convenience have been reported in both regional and international studies, highlighting accessibility as one of the most modifiable determinants of female donation (16, 17).

Collectively, these findings indicate that Saudi women's low participation in blood donation results from the interaction of perceived health vulnerability, cultural beliefs, and limited accessibility rather than from lack of motivation or social responsibility. This interpretation is consistent with national and international evidence emphasizing that facilitation-based interventions are more effective than awareness campaigns alone (6, 11). Strategies such as mobile donation units, workplace and community-based drives, flexible donation hours, and structured post-deferral counseling may therefore be particularly effective in translating willingness into action.

Beyond donor recruitment, increasing female participation has broader clinical and public health implications. Expanding the female donor pool enhances the resilience and diversity of the blood supply and may contribute to improved transfusion outcomes. Emerging evidence suggests that donor–recipient sex matching may influence post-transfusion morbidity and mortality, further underscoring the importance of adequate female donor representation (19, 20). Addressing barriers to female donation is thus relevant not only to equity but also to transfusion safety and effectiveness.

Several limitations should be considered when interpreting these findings. The cross-sectional design precludes causal inference, and reliance on self-reported data introduces potential recall and social desirability bias. The online survey approach may underrepresent women with limited internet access or lower educational levels. Additionally, recruitment through social media platforms may have introduced selection bias, potentially underrepresenting older individuals who are less likely to engage with these platforms. Furthermore, health conditions and deferral history were not verified through clinical records. Nevertheless, the study provides valuable national-level evidence on female-specific barriers to blood donation.

Future research should employ longitudinal and interventional designs to evaluate the effectiveness of targeted strategies such as mobile donation programs, culturally tailored education, and enhanced donor counseling. Inclusion of rural populations and qualitative methodologies may further deepen understanding of contextual influences. Together, these efforts can inform evidence-based policies aimed at creating a gender-inclusive and sustainable blood donation system in Saudi Arabia.

Although Saudi women demonstrate strong altruistic attitudes toward blood donation, actual participation remains low. This study shows that the gap between willingness and donation behavior is primarily driven by health-related uncertainty, persistent cultural misconceptions, and structural barriers rather than lack of motivation. Misunderstandings related to menstruation, anemia, and perceived physical weakness, together with prior deferrals and uncertainty about eligibility, contribute to self-exclusion from donation. In addition, limited accessibility, time constraints, and insufficient availability of mobile donation services further restrict participation. Addressing these barriers requires a shift toward facilitation-focused strategies, including culturally sensitive education, clear communication regarding eligibility and deferral, and improved access through mobile and community-based donation initiatives. Enhancing female participation is essential for strengthening the sustainability and inclusiveness of the national blood supply.

## Data Availability

The raw data supporting the conclusions of this article will be made available by the authors, without undue reservation.
