# Supplementary material for: Evaluation of the cultural, health-related, and structural barriers to blood donation among women in Saudi Arabia: a cross-sectional study
Source: Front Public Health. 2026 Apr 15;14:1806754. doi: 10.3389/fpubh.2026.1806754 (PMC13125013; doi:10.3389/fpubh.2026.1806754)
Supplement: Supplementary file 1 [file Table_1.docx]

Supplementary Material

**Supplementary Questionnaire:**

**Section 1: Eligibility and Demographics**

1. **Do you have a medical condition that permanently prevents you from donating blood**

**(e.g., chronic anemia, heart disease, etc.)?**

- Yes → (End survey for these participants)

- No → (Continue with survey)

2. **Age (select one):**

- 18-25

- 26-35

- 36-45

- 46-55

- More than 55

3. **Marital Status (select one):**

- Single

- Married

4. **Region of Residence (select one):**

- Central (Riyadh, etc.)

- Western (Jeddah, Makkah, etc.)

- Eastern (Dammam, Khobar, etc.)

- Northern

- Southern

5. **Educational Level (select one):**

- Uneducated

- High school

- Bachelor’s degree

- Postgraduate degree (MSc and/or PhD)

6. **Occupation (select one):**

- Student

- Employed

- Self-employed

- Unemployed

7. Household Income (select one):

- Less than 5,000 SAR

- 5,000 – 10,000 SAR- 10,000 – 20,000 SAR

- More than 20,000 SAR

8. **Number of children**

A. 0

B. 1 or 2

C. 3 or 4

D. More than 4

9. **Have you ever donated blood? (select one):**

o Yes

o No

If yes how many…

**Section 2: Blood Donation Knowledge**

10. **Is blood donation harmful to healthy individuals?**

a) No, it is safe and beneficial for most healthy individuals

b) Yes, it causes long-term health problems

c) Yes, it can cause weakness for several months

d) I don’t know

11. **Is the blood stored in blood banks tested and safe for transfusion?**

a) Yes, it is screened for infectious diseases and properly stored

b) No, most of the blood is not tested

c) Only a small number is checked for safety

d) I don’t know

12. **What is the minimum age required to donate blood in Saudi Arabia?**

a) 16 years

b) 17 years

c) 18 years

d) I don’t know

13. **How long can donated red blood cells be safely stored?**

a) 7 days

b) 21 days

c) 42 days

d) 84 days

14. **What is the typical volume of blood collected during a donation?**

a) 200 ml

b) 350 mlc) 450 ml

d) I don’t know

15. **Can pregnant women donate blood?**

a) No, pregnancy is a temporary disqualification for blood donation

b) Yes, at any time during pregnancy

c) Only if the pregnancy is without complications

d) I don’t know

16. **Can a woman donate blood while menstruating?**

a) Yes, if she feels well and meets other eligibility criteria

b) No, menstruation automatically disqualifies donation

c) Yes, but only after the first two days

d) I don’t know

17. **Can cigarette smokers donate blood?**

a) Yes, if they meet all other health requirements

b) No, all smokers are disqualified

c) Only if they haven't smoked in the past 24 hours

d) I don’t know

18. **Can infections or diseases be transmitted through blood transfusion?**

a) Yes, if blood is not properly tested and screened

b) No, there is no risk at all

c) Only in extremely rare cases

d) I don’t know

19. **Do you know the recommended interval between whole blood donations?**

a) Every 2 weeks

b) Every 4 weeks

c) Every 8 weeks

d) I don’t know

20. **How long does the blood donation process usually take?**

a) Less than 10 minutes

b) 15–30 minutes

c) Over 1 hour

d) I don’t know

**Section 3: Attitudes and Beliefs**

21. **Fear of needles prevents me from donating blood.**

□ Strongly Agree □ Agree □ Neutral □ Disagree □ Strongly Disagree

22. **Fear of seeing blood discourages me from donating.**

□ Strongly Agree □ Agree □ Neutral □ Disagree □ Strongly Disagree23. **I avoid donating blood because I’m afraid it will make me weak or sick.**

□ Strongly Agree □ Agree □ Neutral □ Disagree □ Strongly Disagree

24. **Lack of time is the main reason I do not donate blood.**

□ Strongly Agree □ Agree □ Neutral □ Disagree □ Strongly Disagree

25. **I have never donated blood because I don’t know enough about it.**

□ Strongly Agree □ Agree □ Neutral □ Disagree □ Strongly Disagree

26. **There is no specific reason why I haven’t donated blood.**

□ Strongly Agree □ Agree □ Neutral □ Disagree □ Strongly Disagree

27. **Women should not donate blood during menstruation.**

□ Strongly Agree □ Agree □ Neutral □ Disagree □ Strongly Disagree

28. **Women of childbearing age should avoid donating blood.**

□ Strongly Agree □ Agree □ Neutral □ Disagree □ Strongly Disagree

29. **My family or friends influence my decision whether to donate blood.**

□ Strongly Agree □ Agree □ Neutral □ Disagree □ Strongly Disagree

30. **Blood donation is a social responsibility.**

□ Strongly Agree □ Agree □ Neutral □ Disagree □ Strongly Disagree

31. **I am likely to encourage others to donate blood.**

□ Strongly Agree □ Agree □ Neutral □ Disagree □ Strongly Disagree

**Section 4: Health Concerns**

32. **Have you ever been told that you were not eligible to donate blood due to**

**health reasons?**

a) Yes

b) No

33. **yes, what health reason(s) were you told?** *(Select all that apply)*

□ Low hemoglobin or anemia

□ Pregnancy or recent childbirth

□ Use of certain medications

□ Recent infection or illness

□ Other (please specify): ____________

*34.* **Do you currently have any health condition that makes you hesitant to**

**donate blood?**

a) Yes

b) No

35. **If yes, what is your main health concern about donating blood?** *(Open-ended)*

36. **How concerned are you about experiencing side effects (e.g., dizziness,**

**fatigue) after donating blood?**

a) Not at all concerned

b) Slightly concernedc) Moderately concerned

d) Very concerned

e) Extremely concerned

37. **I am worry that donating blood might worsen existing health conditions**

**(such as anemia or low blood pressure).**

a) Strongly agree

b) Agree

c) Neutral

d) Disagree

e) Strongly disagree

38. **I avoid donating blood because I’m not sure if my health status allows it.**

a) Strongly agree

b) Agree

c) Neutral

d) Disagree

e) Strongly disagree

39. **I believe that most women are not healthy enough to donate blood regularly.**

a) Strongly agree

b) Agree

c) Neutral

d) Disagree

e) Strongly disagree

**Section 5: Accessibility and Willingness to Donate**

40. **Do you know where the nearest blood donation center is located?**

a) Yes

b) No

41. **How would you rate the accessibility of blood donation centers in your area?**

a) Very accessible

b) Somewhat accessible

c) Not accessible

d) I don’t know

42. **Would you be willing to donate blood if a mobile donation unit was available**

**near your home or workplace?**

a) Yes, definitely

b) Yes, maybec) No

d) I’m not sure

43. **What would motivate you to donate blood more regularly?** *(Select all that*

*apply)*

□ More awareness about the benefits of blood donation

□ Easier access to blood donation centers

□ Clearer information about health effects of blood donation

□ Support or encouragement from family or friends

□ Financial or material incentives

□ Free health check-ups with each donation

□ Other (please specify): ____________

44. **likely are you to donate blood in the next 6 months?**

a) Very likely

b) Likely

c) Neutral / Not sure

d) Unlikely

e) Very unlikely

45. **I would donate blood more often if the donation process was quicker and**

**more convenient.**

a) Strongly agree

b) Agree

c) Neutral

d) Disagree

e) Strongly disagree

46. **I am willing to donate blood even if I have to travel some distance.**

a) Strongly agree

b) Agree

c) Neutral

d) Disagree

e) Strongly disagree

**Supplemental Table 1. Accessibility and Willingness to Donate Blood Among Females**

| **Accessibility and Willingness to Donate** | **N (%)** |
| --- | --- |
| **Do you know where the nearest blood donation center is located?** |  |
| Yes | 242 (48.1) |
| No | 261 (51.9) |
| **How would you rate the accessibility of blood donation centers in your area?** |  |
| Very accessible | 219 (43.5) |
| Somewhat accessible | 154 (30.6) |
| Not accessible | 23 (4.6) |
| I don’t know | 107 (21.3) |
| **Would you be willing to donate blood if a mobile donation unit was available near your home or workplace?** |  |
| Yes, definitely | 209 (41.6) |
| Yes, maybe | 164 (32.6) |
| No | 40 (8.0) |
| I’m not sure | 90 (17.9) |
| **How likely are you to donate blood in the next 6 months?** |  |
| Very likely | 114 (22.7) |
| Likely | 142 (28.2) |
| Neutral / Not sure | 149 (29.6) |
| Unlikely | 65 (12.9) |
| Very unlikely | 33 (6.6) |
| **I would donate blood more often if the donation process was quicker and more convenient.** |  |
| Strongly agree | 114 (22.7) |
| Agree | 142 (28.2) |
| Neutral | 149 (29.6) |
| Disagree | 65 (12.9) |
| Strongly disagree | 33 (6.6) |
| **I am willing to donate blood even if I have to travel some distance.** |  |
| Strongly agree | 159 (31.6) |
| Agree | 202 (40.2) |
| Neutral | 104 (20.7) |
| Disagree | 26 (5.2) |
| Strongly disagree | 12 (2.4) |
